# The influence of lipoprotein(a) on aortic valve calcification in patients undergoing transcatheter aortic valve replacement

**DOI:** 10.1007/s00392-024-02587-z

**Published:** 2024-12-11

**Authors:** Johanna Bormann, Felix Rudolph, Maximilian Miller, Sara Waezsada, Johannes Kirchner, Sabine Bleiziffer, Kai P. Friedrichs, Volker Rudolph, Tanja K. Rudolph, Muhammed Gerçek

**Affiliations:** 1 Clinic for General and Interventional Cardiology/Angiology, Herz- und Diabeteszentrum Nordrhein-Westfalen, Ruhr-Universität Bochum, Georgstraße 11, 32545 Bad Oeynhausen, Germany; 2 Clinic for Thoracic and Cardiovascular Surgery, Herz- und Diabeteszentrum Nordrhein-Westfalen, Ruhr-Universität Bochum, Bad Oeynhausen, Germany

**Keywords:** TAVR, Lipoprotein(a), Aortic stenosis, Aortic valve, TAVI

## Abstract

**Background:**

Elevated levels of lipoprotein(a) (Lp[a]) have been recognized as substantial risk factors for cardiovascular disease and aortic stenosis (AS). However, the specific role of Lp(a) in promoting aortic valve calcification (AVC) and influencing mortality in elderly, multimorbid patients undergoing transcatheter aortic valve replacement (TAVR) remains unclear and warrants further investigation.

**Methods:**

A retrospective analysis was conducted on all consecutive patients who underwent TAVR between August 2019 and June 2020 at our clinic. Patients with missing data or prior aortic valve replacement were excluded. The study cohort was stratified based on an Lp(a) threshold of 60 mg/dl according to guidelines for lipoprotein apheresis in UK and Germany.^1,2^

**Results:**

In total, 454 patients were included into the analysis. Mean age was 81 ± 6 years and patients presented with a notable cardiovascular risk profile. Lp(a) values ≥ 60 mg/dl were detected in 102 (22.5%) patients, while 352 (77.5%) had Lp(a) values < 60 mg/dl. The median calcium volume of the total cohort was 894.5 [570.8; 1,382.8] mm^2^. No significant difference was observed between the groups (p = 0.83). Furthermore, Lp(a) did not emerge as a statistically significant predictor of calcium levels before TAVR. Notably, male gender (B = 404.11, p < 0.001) and mean trans-valvular pressure gradient (B = 15.64, p < 0.001) were identified as the strongest coefficients within the robust regression analysis. Log-rank tests indicated no prognostic utility of Lp(a) for 30-day all-cause mortality (p = 0.30) or 40 months long-term all-cause mortality (p = 0.60).

**Conclusion:**

Lp(a) might not exert a significant effect on calcification levels or all-cause mortality in patients undergoing TAVR. Despite the study’s highly selected population, these results align with current research, supporting the assumption that the influence of Lp(a) may be confined to the early stages of AS and its progression.

**Supplementary Information:**

The online version contains supplementary material available at 10.1007/s00392-024-02587-z.

## Introduction

Lipoprotein(a) [Lp(a)] has been established as a risk factor for calcification. The expression is genetically determined in up to 90% of cases [1]. Nevertheless, the precise physiological function of Lp(a) remains unclear [2], but associations to pro-atherogenic and pro-inflammatory pathways have been shown previously [3, 4]. Elevated plasma levels of Lp(a) are recognized as an essential independent, causal, and inheritable risk factor for coronary artery disease (CAD) and it’s accelerated progression [2]. There is some evidence that in patients with mild to moderate aortic valve stenosis (AS) elevated Lp(a) levels may also be associated with AS and its progression [5].

Calcific aortic valve disease was estimated to affect 13.32 million people globally in 2021, with an increasing prevalence of AS due to an aging and growing society [6, 7]. Since the five-year mortality of moderate-to-severe and severe AS was shown to be greater than 50% [8], effective therapy is of particular importance. Current treatment strategies involve surgical (SAVR) or transcatheter aortic valve replacement (TAVR) [9].

To date, there are no approved therapeutic options targeting Lp(a) aside from specific lipid apheresis. However, promising results from placebo-controlled, double-blinded Phase III trials indicate that future pharmacological treatments may be viable [10, 11]. Such therapies could potentially serve as therapeutic targets or primary prophylaxis for conditions like coronary artery disease (CAD) and calcific AS. While TAVR and SAVR are efficient and safe treatment options in AS patients, they do not address the underlying cause of the disease.

In addition, the prognostic utility of Lp(a) in advanced stages of AS needs to be sufficiently analyzed [12, 13]. Therefore, this study aims to examine the influence of Lp(a) on aortic valve calcification (AVC) in elderly patients with severe AS who underwent TAVR and evaluate its potential as a prognostic parameter for all-cause mortality.

## Methods

### Study design

**Fig. 1:**
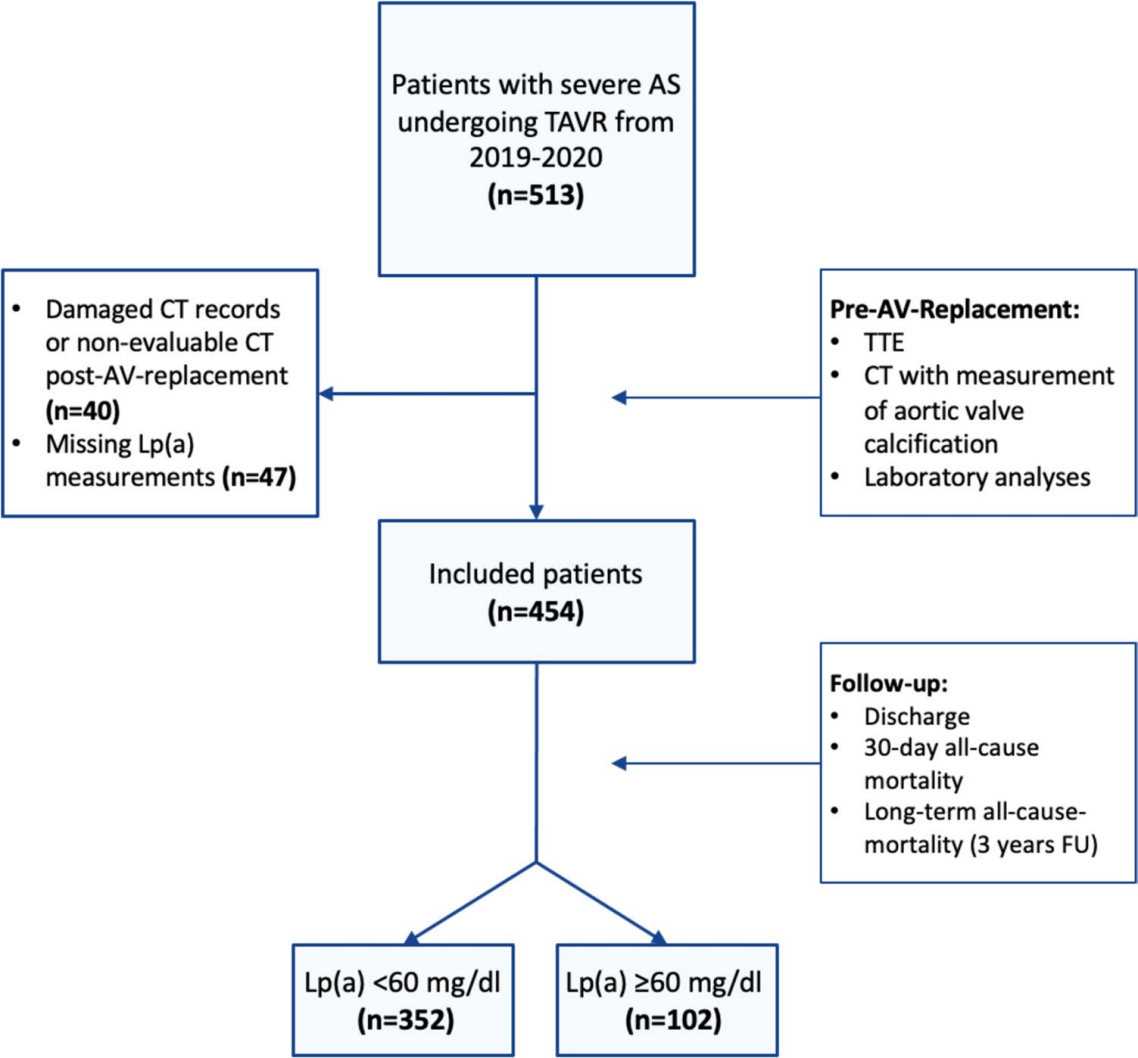
Flow-chart study design

This retrospective observational study was conducted from August 2019 to June 2020 at the Heart and Diabetes Center NRW (HDZ NRW) in Bad Oeynhausen, Germany. The study is an all-comer trial enrolling all patients with severe AS undergoing TAVR. Patients with previous aortic valve replacement were excluded due to difficulties in differentiating calcification on CT scans. Patients with missing Lp(a) measurements or CT records were also excluded. For the analyses, patients were stratified based on a Lp(a) cut-off of 60 mg/dl (Fig. 1). This threshold is also used for determining eligibility for lipoprotein apheresis, currently the only available therapy for cardiovascular risk reduction [14].

### Cholesterol values

All laboratory parameters were measured preoperatively as part of the clinical routine, as is standard practice in our institution. For the determination of Lp(a), an immuno-turbidimetric approach, employing the Alinity c Lp(a) Reagent Kit (Abbott, Abbott Park, IL, USA) was used. Similarly, for the measurement of LDL cholesterol, we employed the direct LDL Reagent Kit Alinity c from Abbott, which determines its concentration by selective dissolution of LDL particles with dye formation. These values also include the LDL components of Lp(a), so additional calculations were carried out to determine the corrected LDL cholesterol values (LDL_cor_). For this purpose, 30% of the respective Lp(a) values were subtracted from the measured LDL value [15].

### CT-scan

The CT scan was typically performed at 100 kV with a slice thickness of 0.5–1 mm. The calcification burden was analyzed using 3Mensio™ Structural Heart software (Pie Medical Imaging, STADT, Netherlands). A virtual basal plane representing the anatomical transition from the left ventricular outflow tract (LVOT) to the aortic root was created using the lowest point of the aortic valve cusps as reference structures. This plane was verified and, if necessary, manually adjusted by an experienced investigator from the heart team blinded to patients’ characteristics. In addition, calcifications of the aortic valve complex (AVC) and left ventricular outflow tract (LVOT) (up to 10 mm below the basal plane) were identified for further TAVR planning. Density values greater than 450 Hounsfield units (HU) were used to determine the calcification burden. This cut-off value was manually refined according to the density of the contrast agent in the LVOT.

### Statistical analysis

Data are reported as absolute numbers and percentages for dichotomous variables, means with standard deviations (SD) for normally distributed continuous variables, and medians with interquartile ranges (IQR) for skewed continuous variables. Group differences were evaluated using Fisher’s exact test for categorical variables, Student’s t-test for normally distributed continuous variables, and the Mann–Whitney U test for non-normally distributed continuous variables. All statistical tests were two-sided, and p-values of < 0.05 were considered significant. Spearman correlations were utilized to describe associations between continuous variables. To further assess the strength of these associations, a regression model was calculated for the most significant correlations. Finally, overall and 30-day mortality were depicted using Kaplan–Meier curves, and survival time analyses were performed to evaluate the associated outcomes. Statistical analyses were conducted using SPSS® Statistics 29 (IBM®, USA) and R 3.6.1 (R Foundation for Statistical Computing, Vienna, Austria).

## Results

### Baseline

A total of 513 patients who presented for TAVR at HDZ NRW Bad Oeynhausen between 2019 and 2020 were initially considered for the study. After excluding 59 patients due to missing data or ViV-procedures, 454 patients were included in the final analysis (Fig. 1). Among these included patients, approximately half were male (243 [53.52%]) with a median age of 82 years (IQR 78–85) and a body-mass index (BMI) of 26.90 [24.07–30.11] kg/m^2^.

Comparison between patients with high Lp(a) levels (≥ 60 mg/dL; n = 102) and those with lower Lp(a) values (n = 352) revealed no significant differences in cardiovascular risk factors or pre-existing conditions, including coronary artery disease (CAD), chronic renal insufficiency, diabetes, previous myocardial infarction (MI), or stroke (Table 1). Additionally, echocardiographic parameters were comparable between both groups, with a median aortic valve area (AVA) of 0.7 [IQR 0.6–0.9] cm^2^, a maximum velocity (Vmax) of 4.01 [IQR 3.53–4.39] m/s, and a mean gradient of 43 [IQR 33–52] mmHg.

**Table 1:** Baseline characteristics

	All	Lp(a) < 60	Lp(a) ≥ 60	p-value
	n = 454	n = 352	n = 102	
Age [median (IQR), [years]	82 (78–85)	82 (78–85)	81 (77.25–85)	0.42
Male gender [n (%)]	243 (53.52)	194 (55.11)	49 (48.04)	0.22
Cardiovascular risk factors				
Arterial hypertension [n (%)]	415 (91.41)	321 (91.19)	94 (92.16)	0.84
Diabetes mellitus [n (%)]	67 (17.77)	49 (17.07)	18 (20)	0.35
Smoking [n (%)]	25 (5.51)	18 (5.11)	7 (6.86)	0.47
BMI [median (IQR), kg/m^2^]	26.9 (24.07–30.11)	26.95 (24.11–30.41)	26.57 (23.89–29.73)	0.46
History				
CAD [n (%)]	254 (55.95)	197 (55.97)	57 (55.88)	> 0.99
1VD [n (%)]	89 (35.04)	68 (34.52)	21 (36.84)	0.78
2VD [n (%)]	73 (28.74)	59 (29.95)	14 (24.56)	0.54
3VD [[n (%)]	92 (36.22)	70 (35.53)	22 (38.6)	0.78
CKD ≥ 2 [n (%)]	239 (52.64)	181 (51.42)	58 (56.86)	0.37
MI [n (%)]	48 (10.57)	36 (10.23)	12 (11.76)	0.72
Stroke [n (%)]	16 (3.52)	13 (3.69)	3 (2.94)	> 0.99
Laboratory analyses				
eGFR [median (IQR), ml/min/1.73m^2^]	58 (43–70)	58 (43–71)	57.5 (43–68.75)	0.32
Creatinine [median (IQR), mg/dl]	1.1 (0.87–1.4)	1.1 (0.87–1.33)	1.1 (0.88–1.4)	0.51
HbA1c [median (IQR), %]	5.7 (5.4–6.2)	5.7 (5.4–6.3)	5.7 (5.4–6.07)	0.49
Lp(a) [median (IQR), mg/dl]	16.5 (7–53)	11 (6–23)	92 (75–113)	** < 0.001**
Cholesterol [median (IQR), mg/dl]	178.5 (146–211.75)	175.5 (144–207)	193.5 (154.5–221.75)	**0.007**
LDL-C [median (IQR), mg/dl]	106 (81–137)	104 (80–134.5)	116.5 (85.5–150.25)	**0.015**
LDL-C corrected [median (IQR), mg/dl]	95.7 (70.8–128.1)	98 (74.2–128.7)	85.45 (58.12–117.35)	**0.005**
HDL-C [median (IQR), mg/dl]	51 (42–63)	50.5 (42–60)	56 (44–66.5)	**0.014**
Triglycerides [median (IQR), mg/dl]	112 (83–144)	112 (83–148)	112 (80–133.75)	0.33
Statin [n (%)]	289 (63.66)	220 (62.5)	69 (67.65)	0.35
Echocardiographic parameter of aortic valve				
AVA [median (IQR), cm^2^]	0.7 (0.6–0.9)	0.7 (0.6–0.9)	0.7 (0.6–0.9)	0.89
AVA/BSA [median (IQR), cm^2^/m^2^]	0.4 (0.34–0.47)	0.4 (0.34–0.47)	0.4 (0.33–0.48)	0.72
Vmax [median (IQR), m/s]	4.01 (3.53–4.39)	4.06 (3.54–4.43)	3.9 (3.36–4.29)	0.19
ΔPm [median (IQR), mmHg]	43 (33–52)	42 (32–51.25)	47 (35.5–54.5)	0.24
CT measurement of aortic valve calcification				
Overall calcification [median (IQR), mm^3^]	894.5 (571–1381.75)	891.5 (577.75–1387.25)	942.5 (548.75–1343)	0.83
AVC [median (IQR), mm^3^]	829 (528.25–1236.75)	824.5 (532–1247.5)	869.5 (457.25–1161)	0.83
LVOT [median (IQR), mm^3^]	23 (1–116.25)	23 (1–114.75)	24 (2–120)	0.55

Bold front illustrates significant values

*CAD* coronary artery disease, *VD* vessel disease, *CKD* chronic kidney disease, *MI* myocardial infarction, *eGFR* estimated glomerular filtration rate, *LDL-C* low-density lipoprotein cholesterol, *HDL-C* high-density lipoprotein cholesterol, *AVA* aortic valve area, *Vmax* maximum velocity, *ΔPm* mean gradient, *AVC* aortic valve cusps, *LVOT* left ventricular outflow tract

### Lipid profile

Among the 454 patients, 102 (22.47%) had an Lp(a) value ≥ 60 mg/dL, while 352 (77.53%) had Lp(a) values below this cut-off. One-third of the patients had Lp(a) values ≥ 30 mg/dL (n = 167 [36.80%]), and 55 patients (12.10%) had values ≥ 90 mg/dL.

In the group with elevated Lp(a) values (≥ 60 mg/dL), total cholesterol (p = 0.007), LDL cholesterol (p = 0.015), and HDL cholesterol (p = 0.014) were significantly higher (Table 1). Due to the higher proportion of Lp(a), the corrected LDL cholesterol levels were higher in the Lp(a) < 60 mg/dl group (LDL-C_corr_ 98.00 [74.20–128.70] mg/dl vs. 85.45 [58.12–117.35] mg/dl; p = 0.005). There was no significant difference in triglycerides. In addition, approximately two-thirds of patients in both groups were on statins (62.50% vs. 67.65%; p = 0.35).

### Calcification

**Fig. 2:**
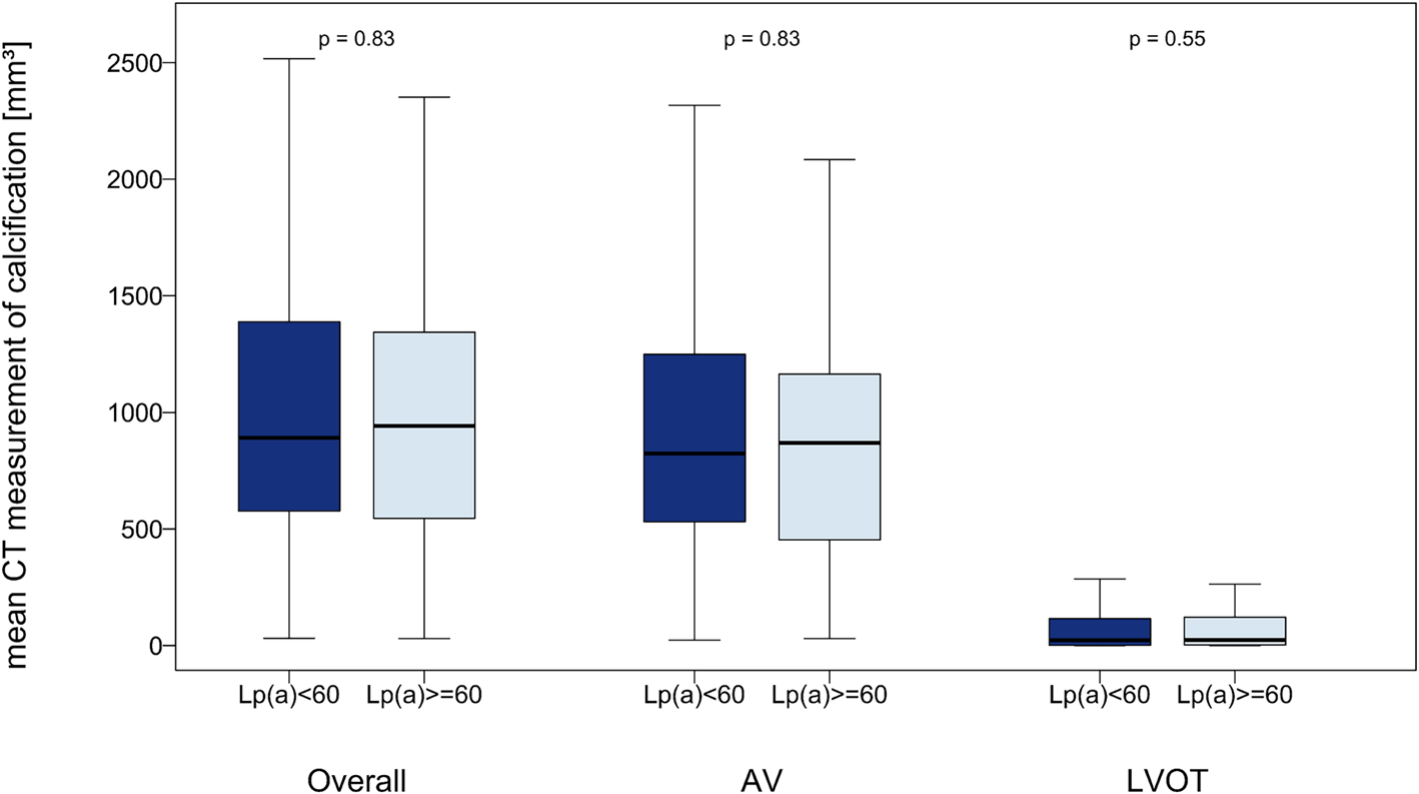
CT-Measurement of Aortic valve calcification in mm^3^ in different localizations (overall, ACV and LVOT) divided based on Lp(a) values (< 60 mg/dl: dark blue vs. > 60 mg/dl: sky blue)

CT scans revealed a total aortic valve calcification volume of 894.50 [571.00–1381.75] mm^3^. There was no significant difference in calcification between the high Lp(a) group (≥ 60 mg/dL) and the lower Lp(a) group (p = 0.83), both in the aortic valve cusps (AVC) (824.50 [532.00–1247.50] mm^3^ and 869.50 [457.25–1161.00] mm^3^; p = 0.83), which accounted for the majority of the total calcification, and in the left ventricular outflow tract (LVOT) (23.00 [1.00–114.75] mm^3^ and 24.00 [2.00–120.00] mm^3^; p = 0.55) (Fig. 2).

Spearman correlation analysis showed no significant association between Lp(a) levels and total calcification (ρ = −0.037; p = 0.43), AVC calcification (ρ = −0.031; p = 0.51), or LVOT calcification (ρ = −0.0023; p = 0.96).

However, there was a low correlation between overall and AVC calcification with the maximum velocity (Vmax) across the aortic valve (_Overall_ ρ = 0.39; p < 0.0001 and _AVC_ ρ = 0.37; p < 0.0001) as well as the mean gradient ΔPm (_Overall_ ρ = 0.43; p < 0.0001 and _AVC_ ρ = 0.45; p < 0.0001). Furthermore, there was a negative linear relationship with the aortic valve area (AVA) and AVA indexed to body surface area (AVA/BSA) according to DuBois (AVA: _Overall_ ρ = −0.21; p < 0.0001 and _AVC_ ρ = −0.23; p < 0.0001 and AVA/BSA: _Overall_ ρ = −0.31; p < 0.0001 and _AVC_ ρ = −0.34; p < 0.0001). In the regression model ΔPm (B = 17.35 [95% CI 12.4; 23.01], p = 0.01), AVA/BSA (B = −646.71 [95% CI −1279.91; −126.98], p = 0.025), and male gender (B = 441.21 [95% CI 320.46; 575.68], p = 0.01) were statistically significant predictors for calcification volume of the entire aortic valve.

### Subgroup analysis

Given that the regression analysis indicated a correlation between male gender and elevated calcification levels with higher calcification volumes overall (male 1101 [IQR 781.5—1553] mm3 and female 654 [IQR 426—1046] mm^3^), a subgroup analysis was conducted to eliminate the potential for confounding factors associated with gender-specific differences. This analysis of male and female patients revealed no significant differences in AVC and LVOT calcification. Overall, there were no significant differences in cardiovascular risk factors, echocardiographic parameters, and mortality between patients with high (≥ 60 mg/dl) and low (< 60 mg/dl) Lp(a) levels in the female or the male subgroup.

Additionally, an analysis of the data using Lp(a) thresholds of 30 mg/dl and 90 mg/dl was performed. Similarly, no significant differences were observed in calcification burden or echocardiographic parameters (Supplemental Table 1 and Table 2).

**Table 2:** Discharge characteristics with echocardiographic parameter and Events post-TAVI

	All	Lp(a) < 60	Lp(a) ≥ 60	p-value
	n = 454	n = 352	n = 102	
Duration of hospitalization [median (IQR), days]	9 (7–12)	9 (7–12)	9 (7–11.75)	0.81
ΔPp [median (IQR), mmHg]	13 (8–18)	12 (8–18)	14 (8–18)	0.50
ΔPm [median (IQR), mmHg]	7 (4–10)	7 (4–10)	8 (5–10)	0.25
PVL [n (%)]	187 (41.74)	148 (42.53)	39 (39)	0.57
Minimal or mild PVL [n (%)]	175 (39.15)	137 (39.48)	38 (38)	0.82
Moderate PVL [n (%)]	11 (2.46)	10 (2.88)	1 (1)	0.47
PM-Rhythm [n (%)]	83 (18.78)	62 (18.08)	21 (21.21)	0.56
Stroke [n (%)]	10 (2.2)	8 (2.27)	2 (1.96)	> 0.99
MI [n (%)]	1 (0.22)	1 (0.28)	0 (0)	> 0.99

*ΔPp* peak gradient, *ΔPm* mean gradient, *PVL* paravalvular leakage, *PM-Rhythm* pacemaker-rhythm, *MI*  myocardial infarction

### Post-interventional course and mortality

Patients stayed in the hospital for 9 [6–11] days following TAVR with no statistically significant differences between the Lp(a) groups (p = 0.81). Post-TAVI echocardiographic assessment revealed slightly higher gradients in patients with elevated Lp(a) levels (≥ 60 mg/dl), with a mean gradient (ΔPmean) of 8 mmHg (IQR 5–10) and a peak gradient (ΔPpeak) of 14 mmHg (IQR 8–18); however, these differences were not statistically significant (p = 0.50 and p = 0.25, respectively). Similarly, at discharge, the proportion of patients with paravalvular leaks (PVL) was comparable across Lp(a) groups (p = 0.57). The distribution of PVL severity grades also showed no significant differences between the Lp(a) groups. Furthermore, our analysis demonstrated a consistent prevalence of patients with a pacemaker rhythm at discharge across both groups (18.1% vs. 21.2%; p = 0.56), as well as stroke and myocardial infarction.

**Fig. 3:**
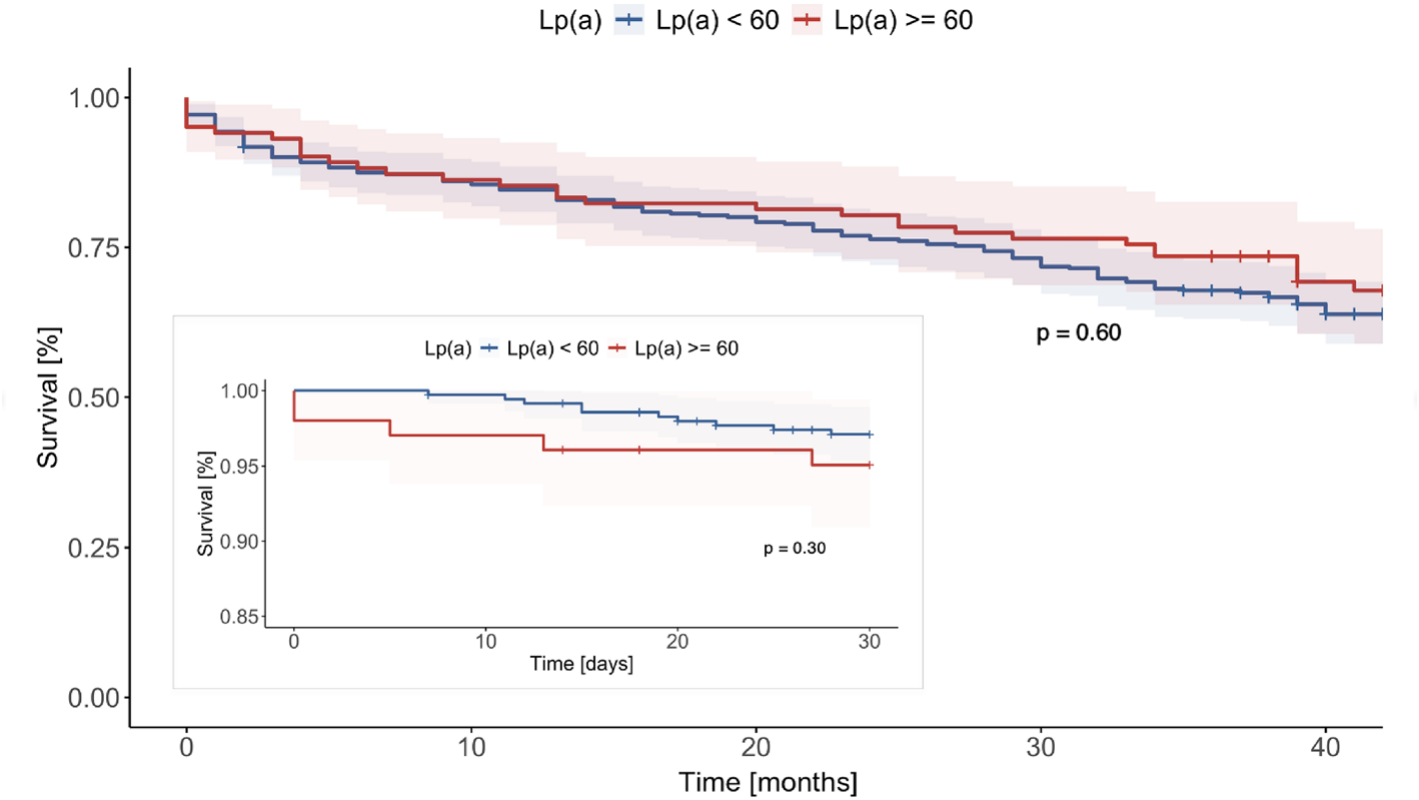
Kaplan–Meier-Curves with corresponding confidence interval of TAVR patients divided on Lp(a) levels (in red patients with an Lp(a)-level ≥ 60 mg/dl and in blue patients with Lp(a)-level < 60 mg/dl). The small box shows an enlarged excerpt with the 30-day mortality

During the initial 30 days, a 30-day all-cause mortality of 3.3% was observed. The 30-day survival rates did not differ between the groups stratified by their Lp(a) levels (97.2% vs. 95.1%; p = 0.30). The overall follow-up period spanned 38 [28.25–48.00] months with a mortality rate of 37.2%. Furthermore, during the follow-up period of nearly 4 years (see Fig. 3), no significant difference in survival rates has been observed between both groups (Lp(a)_<60 mg/dL_ 62.22% and Lp(a)_≥60 mg/dL_ 64.71%; p = 0.60).

## Discussion

Lp(a) is considered an independent risk factor for the occurrence of cardiovascular diseases such as coronary artery disease (CAD), arteriosclerosis (AS), and myocardial infarction [1, 5, 16]. Nevertheless, its significance, especially in elderly patients, is sometimes controversially discussed in the literature [17–20].

Our study is the first to show no effect of Lp(a) on aortic valve calcification and prognosis in a general population of patients with severe AS undergoing TAVR. Our main findings are as follows: first, elevated Lp(a) levels showed no impact on the degree of aortic valve calcification in the studied TAVR cohort. Second, these results were also reflected in the subgroup analysis of male and female patients. Third, an elevated Lp(a) value ≥ 60 mg/dl demonstrated no prognostic influence on all-cause mortality at 30 days and 3 years post-TAVR. These results provide an essential addition to existing knowledge and raise questions about the role of Lp(a) in aortic valve calcification.

### Study cohort

The study cohort of 454 patients comprised multimorbid TAVR patients regarding baseline data and preexisting conditions. Consistent with the findings of Mauri et al. (15,344 patients from five German TAVR centers), the patients exhibited a similarly high age (average 81 years) and a pronounced cardiovascular risk profile [21]. The echocardiographic parameters obtained, with a ΔPm of 44.15 ± 15.72 mmHg and an AVA of 0.75 ± 0.20 cm^2^, align with other TAVR and SVAR registries [22, 23]. The restriction of the valve opening area is attributed to complex fibrotic and calcific remodeling [9, 24]. Consequently, it is not surprising that this study also demonstrated a correlation between severe aortic valve calcification and higher Vmax and delta Pm. Furthermore, our finding indicating an association between male gender and higher levels of calcification are consistent with the ECS/EACTs guidelines cut-off values for men with calcified aortic stenosis, which specify higher Agatston thresholds for men in native CT scans [9]. The method used in this study to evaluate the contrast-enhanced CT images with the 3Mensio™ software was also employed in the TAVR registry by Kim et al. [25] Initial studies have indicated that the results of calcification evaluation using 3Mensio are potentially comparable to those obtained with other software, such as CVI 42™ (Circle Cardiovascular Imaging Inc., Canada) or SyngoVia™ (Siemens Healthcare GmbH, Germany) [26]. Nevertheless, the lack of standardized protocols for contrast-enhanced CT images may continue to hinder comparability [25].

### Lp(a) as cardiovascular risk factor in aortic stenosis

The prognostic utility of Lp(a) in advanced stages of AS is not well established, and there are currently no standardized cut-off levels for Lp(a) [12, 13]. Different guidelines and consensus statements suggest varying cut-offs for elevated Lp(a), ranging from 30 to 90 mg/dL [27]. However, lipid apheresis guidelines specify a 60 mg/dL cut-off [1, 14]. Given that lipid apheresis is presently the only therapeutic option for reducing Lp(a) levels, we adopted this cut-off in our study. In general, Lp(a) levels vary widely, from almost 0 to > 200 mg/dl, [28] with values as low as approximately 20 mg/dl associated with a steadily increasing risk of cardiovascular diseases such as myocardial infarction, stroke, or aortic valve stenosis. [29–31] Additionally, there is a 2- to fourfold median difference in plasma Lp(a) levels among different ethnic groups [27], with about 20% of the Caucasian population having Lp(a) levels > 50 mg/dl [32], a prevalence that is higher in African ethnic groups and lower in Asian ethnic groups [33, 34]. The Lp(a) distribution in this study resembles previously described distributions [35, 36], but there is a higher proportion of participants with values ≥ 50 mg/dl (26.0%), matching the Lp(a) distributions observed in patient groups with a high cardiovascular burden [36].

Elevated Lp(a) levels are associated with an increased risk of cardiovascular disease (CVD) and a higher severity of coronary artery disease (CAD) [1, 37]. In patients with CAD, those with elevated Lp(a) levels above 150 nmol/l were more likely to have severe CAD that is more difficult to treat [38]. However, in this older and multimorbid TAVR cohort, no differences were found in the proportion of patients with CAD between the groups with an Lp(a) cut-off of 60 mg/dl, whereas the proportion of patients with CAD is in the upper range compared to other TAVR registries [21, 39]. These results corroborate the assertion by Cicec et al. that Lp(a) exerts a role as a risk factor for CAD primarily in younger patients (aged < 65 years) and appears to lose significance with age [17]. Conversely, studies have demonstrated that elevated Lp(a) levels remain an independent risk factor for coronary heart disease in slightly older patients with a median age of 71 years. [35] Nevertheless, the clinical benefit of a reduction in elevated Lp(a) remains unclear in these studies [35, 40]. This is of particular interest in regard to the fact that several phase III trials for a specific drug-based reduction of Lp(a) are already underway [10, 11].

There is limited evidence on the direct or quantitative impact of Lp(a) on aortic valve calcification (AVC) [41]. Our analysis shows that despite the known association of Lp(a) with various cardiovascular diseases, no significant influence on AVC in elderly patients with preexistent severe AS could be observed. Lp(a)’s influence might be restricted to the early stages of calcification (initiation phase), losing significance as calcification progresses [41]. In the younger patients (mean age 58 years) from the ASTRONOMER trial with mild to moderate stenosis and elevated Lp(a), a faster disease progression with Vmax increase could be described [5]. This finding is consistent with the results reported by Vongpromek et al. [42]. Whereas a faster progression of ΔPm and Vpeak in patients with higher Lp(a) values were not significant in the PROGRESSA [43] and SALTIRE2 [44] trial [45]. It is conceivable that the impact of Lp(a) in AS is limited to the younger patients with AVC, who are more likely to be included in SAVR cohorts [46]. In a smaller cohort of 210 patients, Farina et al. [47] demonstrated that bioprosthetic valves (70% SVAR) exhibited faster degeneration in the presence of elevated Lp(a) levels. However, it remains unclear whether this finding can be extrapolated to the typically older TAVR patient population.

### Prognostic value of Lp(a) in TAVR

In the highly debated field regarding the influence of Lp(a) in multimorbid, elderly patients with advanced severe aortic stenosis, this study found no evidence of a prognostic impact on all-cause mortality. Our results showed no significant differences in 30-day or long-term all-cause mortality, with a mean follow-up time of 38 months. Similar to this study, there are 30-day mortality rates of 2.1% in other German TAVR registries [21]. However, there are no other existing analyses on the impact of Lp(a) on mortality rates in TAVR patients. The observation that both groups exhibit a similarly high risk may be influenced by the bias that Lp(a) is recognized as a cardiovascular risk factor, prompting these patients to attend regular follow-ups with their cardiologists. However, it should also be noted that most TAVR patients are routinely subjected to close, structured follow-up care after the procedure, irrespective of their Lp(a) levels.

Furthermore, no significant differences were observed in the echocardiographic parameters at discharge, including ΔPmean, ΔPpeak, and the prevalence of PVL, between patients with elevated Lp(a) levels (> 60 mg/dl) and those with lower Lp(a) levels. The extent and distribution of calcification are recognized as factors influencing PVL post-TAVI [48]. Since Lp(a) did not show any impact on calcification in CT imaging in our study, this may partly explain the lack of differences in PVL prevalence between the groups.

### Limitations

This study has several limitations. First, it is a retrospective observational study with only 454 patients, which means that potential biases leading to overestimation or underestimation of effects cannot be excluded. Second, patients from a single center and a very specific multimorbid patient cohort were included, which might limit the generalizability of the results to other populations due to possible additional factors not explicitly captured here. However, despite being a single-center cohort, the study population demonstrates good comparability with other larger multicenter TAVR registries [21].

Additionally, the lack of uniform thresholds and measurement methods for Lp(a) and the absence of standardized procedures for assessing aortic valve calcification in contrast-enhanced CT scans further restrict the generalizability of the findings.

## Conclusion

Our study presents no significant role of Lp(a) in aortic valve calcification or morality in a elderly TAVR cohort. The calcification volume of aortic cusps and LVOT was nearly similar in TAVR patients divided on Lp(a) levels. In addition, no increase in mortality was observed at high Lp(a) levels. Our results suggest that the predictive significance of Lp(a) for aortic valve calcification and treatment outcomes after TAVR may be lower than previously assumed. This finding may indicate that different cardiovascular pathologies with complex interactions attenuate the effects of Lp(a) in severe AS patients undergoing TAVR.

## Supplementary Information

Below is the link to the electronic supplementary material.

Supplementary file1 (DOCX 22 KB)

## Data Availability

The data that support the findings of this study are available from the corresponding author upon reasonable request.

## Abbreviations

ACV: Aortic valve cusps
AS: Aortic stenosis
AVA: Aortic valve area
AVA/BSA: Aortic valve area indexed to body surface area
B: Regression coefficient
BSA: Body surface area
CAD: Coronary artery disease
CKD: Chronic kidney disease
CT: Computed tomography
CVD: Cardiovascular disease
ΔPm: Mean gradient
ΔPp: Peak gradient
PVL: Paravalvular leakage
eGFR: Estimated glomerular filtration rate
HbA1c: Glycated hemoglobin
HDL-C: High-density lipoprotein cholesterol
HU: Hounsfield units
IQR: Interquartile range
LDL: Low-density lipoprotein
LDL-C: Low-density lipoprotein cholesterol
LDL_cor_: Corrected low-density lipoprotein cholesterol
Lp(a): Lipoprotein(a)
LVOT: Left ventricular outflow tract
MI: Myocardial infarction
SD: Standard deviation
SAVR: Surgical aortic valve replacement
TAVR: Transcatheter aortic valve replacement
TTE: Transthoracic echocardiography
Vmax: Maximum velocity
