# Correction: The influence of lipoprotein(a) on aortic valve calcification in patients undergoing transcatheter aortic valve replacement

**DOI:** 10.1007/s00392-024-02592-2

**Published:** 2025-01-07

**Authors:** Johanna Bormann, Felix Rudolph, Maximilian Miller, Sara Waezsada, Johannes Kirchner, Sabine Bleiziffer, Kai P. Friedrichs, Volker Rudolph, Tanja K. Rudolph, Muhammed Gerçek

**Affiliations:** 1 Clinic for General and Interventional Cardiology/Angiology, Herz- und Diabeteszentrum Nordrhein-Westfalen, Ruhr-Universität Bochum, Georgstraße 11, 32545 Bad Oeynhausen, Germany; 2 Clinic for Thoracic and Cardiovascular Surgery, Herz- und Diabeteszentrum Nordrhein-Westfalen, Ruhr-Universität Bochum, Bad Oeynhausen, Germany

Correction: Clinical Research in Cardiology 10.1007/s00392-024-02587-z

When this article was first published, it had been forgotten to mention that the authors Johanna Bormann and Felix Rudolph do share the first authorship.

The original article has been updated.

