# Supplementary material for: The influence of lipoprotein(a) on aortic valve calcification in patients undergoing transcatheter aortic valve replacement
Source: Clin Res Cardiol. 2024 Dec 11;114(3):395–404. doi: 10.1007/s00392-024-02587-z (PMC11913987; doi:10.1007/s00392-024-02587-z)
Supplement: Supplementary file 1 — Supplementary file1 (DOCX 22 KB) [file 392_2024_2587_MOESM1_ESM.docx]

## **Supplemental material**

|  | **All** | **Lp(a) <30** | **Lp(a) ≥30** | **p-value** |
| --- | --- | --- | --- | --- |
|  | **n = 454** | **n = 287** | **n = 167** |  |
| Age [median (IQR), [years] | 82 (78-85) | 82 (78-85) | 81 (78-85) | 0.52 |
| Male Gender [n (%)] | 243 (53.52) | 158 (55.05) | 85 (50.9) | 0.44 |
| **Cardiovascular Risk Factors** |  |  |  |  |
| Arterial Hypertension [n (%)] | 415 (91.41) | 261 (90.94) | 154 (92.22) | 0.73 |
| Diabetes mellitus [n (%)] | 67 (17.77) | 40 (16.95) | 27 (19.15) | 0.58 |
| Smoking [n (%)] | 25 (5.51) | 15 (5.23) | 10 (5.99) | 0.83 |
| BMI [median (IQR), kg/m^2^] | 26.9 (24.07-30.11) | 26.93 (24.11-30.39) | 26.83 (23.91-29.83) | 0.57 |
| **History** |  |  |  |  |
| CAD [n (%)] | 254 (55.95) | 163 (56.79) | 91 (54.49) | 0.69 |
| 1VD [n (%)] | 89 (35.04) | 57 (34.97) | 32 (35.16) | 0.90 |
| 2VD [n (%)] | 73 (28.74) | 50 (30.67) | 23 (25.27) | 0.35 |
| 3VD [[n (%)] | 92 (36.22) | 56 (34.36) | 36 (39.56) | 0.63 |
| CKD ≥ 2 [n (%)] | 239 (52.64) | 145 (50.52) | 94 (56.29) | 0.24 |
| MI [n (%)] | 48 (10.57) | 33 (11.5) | 15 (8.98) | 0.43 |
| Stroke [n (%)] | 16 (3.52) | 11 (3.83) | 5 (2.99) | 0.79 |
| **Laboratory analyses** |  |  |  |  |
| eGFR [median (IQR), ml/min/1.73m^2^] | 58 (43-70) | 59 (43.5-71.5) | 56 (42.5-68) | 0.15 |
| Creatinine [median (IQR), mg/dl] | 1.1 (0.87-1.4) | 1.1 (0.87-1.4) | 1.1 (0.9-1.4) | 0.17 |
| HbA1c [median (IQR), %] | 5.7 (5.4-6.2) | 5.7 (5.4-6.3) | 5.7 (5.4-6.1) | 0.71 |
| Lp(a) [median (IQR), mg/dl] | 16.5 (7-53) | 8 (4-15) | 71 (45-97.5) | <0.001 |
| Cholesterol [median (IQR), mg/dl] | 178.5 (146-211.75) | 172 (145-204.5) | 186 (149.5-225) | 0.013 |
| LDL-C [median (IQR), mg/dl] | 106 (81-137) | 103.5 (80-131) | 114 (83-146.5) | 0.03 |
| LDL-C corrected [median (IQR), mg/dl] | 95.7 (70.8-128.1) | 99.65 (76.4-128.75) | 88.2 (60.1-125.5) | 0.006 |
| HDL-C [median (IQR), mg/dl] | 51 (42-63) | 50 (42-59) | 54 (43.5-65) | 0.039 |
| Triglycerides [median (IQR), mg/dl] | 112 (83-144) | 113 (84-148) | 109.5 (80-136.25) | 0.19 |
| Statin [n (%)] | 289 (63.66) | 186 (64.81) | 103 (61.68) | 0.54 |
| **Echocardiographic parameter BL** |  |  |  |  |
| AVA [median (IQR), cm^2^] | 0.7 (0.6-0.9) | 0.7 (0.6-0.83) | 0.7 (0.6-0.9) | 0.41 |
| AVA/BSA [median (IQR), cm^2^/m^2^] | 0.4 (0.34-0.47) | 0.4 (0.33-0.47) | 0.41 (0.34-0.49) | 0.25 |
| Vmax [median (IQR), m/s] | 4.01 (3.53-4.39) | 4.01 (3.57-4.42) | 4 (3.43-4.37) | 0.50 |
| Δ Pm [median (IQR), mmHg] | 43 (33-52) | 42 (32.75-52) | 45 (33-52.5) | 0.62 |
| **CT measurement of calcification** |  |  |  |  |
| Overall calcification [median (IQR), mm^3^] | 894.5 (571-1381.75) | 908 (581-1486) | 881 (538-1260) | 0.18 |
| AVC [median (IQR), mm^3^] | 829 (528.25-1236.75) | 850 (539-1280) | 814 (461.5-1132.5) | 0.22 |
| LVOT [median (IQR), mm^3^] | 23 (1-116.25) | 23 (1-131) | 23 (1-95) | 0.62 |
| **Discharge** |  |  |  |  |
| PPG [median (IQR), mmHg] | 13 (8-18) | 12 (8-17) | 14 (8-19) | 0.13 |
| MPG [median (IQR), mmHg] | 7 (4-10) | 6 (4-9) | 8 (5-10.75) | 0.11 |
| PVL [n (%)] | 187 (41.74) | 118 (41.55) | 69 (42.07) | >0.99 |
| PM-Rhythm [n (%)] | 83 (18.78) | 51 (18.21) | 32 (19.75) | 0.71 |
| Stroke [n (%)] | 10 (2.2) | 7 (2.44) | 3 (1.8) | 0.75 |
| MI [n (%)] | 1 (0.22) | 1 (0.35) | 0 (0) | >0.99 |
| **Follow up** |  |  |  |  |
| Follow up time [median (IQR), months] | 38 (28.25-48) | 38 (26.5-48) | 39 (31-48) | 0.33 |
| Death [n (%)] | 169 (37.22) | 112 (39.02) | 57 (34.13) | 0.32 |

|  | **All** | **Lp(a) <90** | **Lp(a) ≥90** | **p-value** |
| --- | --- | --- | --- | --- |
|  | **n = 454** | **n = 399** | **n = 55** |  |
| Age [median (IQR), [years] | 82 (78-85) | 82 (78-85) | 81 (78-85) | 0.68 |
| Male Gender [n (%)] | 243 (53.52) | 219 (54.89) | 24 (43.64) | 0.15 |
| **Cardiovascular Risk Factors** |  |  |  |  |
| Arterial Hypertension [n (%)] | 415 (91.41) | 367 (91.98) | 48 (87.27) | 0.3 |
| Diabetes mellitus [n (%)] | 67 (17.77) | 56 (17.23) | 11 (21.15) | 0.23 |
| Smoking [n (%)] | 25 (5.51) | 19 (4.76) | 6 (10.91) | 0.10 |
| BMI [median (IQR), kg/m^2^] | 26.9 (24.07-30.11) | 26.99 (24.22-30.39) | 26.22 (23.3-29.4) | 0.10 |
| **History** |  |  |  |  |
| CAD [n (%)] | 254 (55.95) | 226 (56.64) | 28 (50.91) | 0.47 |
| 1VD [n (%)] | 89 (35.04) | 78 (34.51) | 11 (39.29) | >0.99 |
| 2VD [n (%)] | 73 (28.74) | 65 (28.76) | 8 (28.57) | 0.85 |
| 3VD [[n (%)] | 92 (36.22) | 83 (36.73) | 9 (32.14) | 0.59 |
| CKD ≥ 2 [n (%)] | 239 (52.64) | 210 (52.63) | 29 (52.73) | >0.99 |
| MI [n (%)] | 48 (10.57) | 45 (11.28) | 3 (5.45) | 0.25 |
| Stroke [n (%)] | 16 (3.52) | 15 (3.76) | 1 (1.82) | 0.71 |
| **Laboratory analyses** |  |  |  |  |
| eGFR [median (IQR), ml/min/1.73m^2^] | 58 (43-70) | 58 (43-70) | 58 (43-70.5) | 0.80 |
| Creatinine [median (IQR), mg/dl] | 1.1 (0.87-1.4) | 1.1 (0.88-1.4) | 1.1 (0.86-1.4) | 0.86 |
| HbA1c [median (IQR), %] | 5.7 (5.4-6.2) | 5.7 (5.4-6.2) | 5.6 (5.4-6) | 0.21 |
| Lp(a) [median (IQR), mg/dl] | 16.5 (7-53) | 13 (6-33) | 112 (99.5-134) | <0.001 |
| Cholesterol [median (IQR), mg/dl] | 178.5 (146-211.75) | 177 (145-208) | 198 (157-224.5) | 0.013 |
| LDL-C [median (IQR), mg/dl] | 106 (81-137) | 104.5 (81-135.75) | 121 (87-153.5) | 0.024 |
| LDL-C corrected [median (IQR), mg/dl] | 95.7 (70.8-128.1) | 97.05 (73.12-128.55) | 83.1 (55.25-116.8) | 0.013 |
| HDL-C [median (IQR), mg/dl] | 51 (42-63) | 51 (42-61) | 61 (44.5-69.5) | 0.005 |
| Triglycerides [median (IQR), mg/dl] | 112 (83-144) | 112 (83-148) | 112 (83-133.5) | 0.48 |
| Statin [n (%)] | 289 (63.66) | 254 (63.66) | 35 (63.64) | >0.99 |
| **Echocardiographic parameter BL** |  |  |  |  |
| AVA [median (IQR), cm^2^] | 0.7 (0.6-0.9) | 0.7 (0.6-0.9) | 0.7 (0.65-0.9) | 0.80 |
| AVA/BSA [median (IQR), cm^2^/m^2^] | 0.4 (0.34-0.47) | 0.4 (0.33-0.47) | 0.41 (0.34-0.48) | 0.38 |
| Vmax [median (IQR), m/s] | 4.01 (3.53-4.39) | 4.04 (3.57-4.42) | 3.71 (3.3-4.04) | 0.036 |
| Δ Pm [median (IQR), mmHg] | 43 (33-52) | 42 (32-51) | 47.5 (37.25-57.75) | 0.14 |
| **CT measurement of calcification** |  |  |  |  |
| Overall calcification [median (IQR), mm^3^] | 894.5 (571-1381.75) | 895 (581-1397) | 868 (522-1240) | 0.22 |
| AVC [median (IQR), mm^3^] | 829 (528.25-1236.75) | 842 (534.5-1248.5) | 769 (445-1100.5) | 0.19 |
| LVOT [median (IQR), mm^3^] | 23 (1-116.25) | 23 (1-118) | 18 (1-91.5) | 0.72 |
| **Discharge** | 2 |  |  |  |
| PPG [median (IQR), mmHg] | 13 (8-18) | 12.5 (8-18) | 13 (8-17) | 0.75 |
| MPG [median (IQR), mmHg] | 7 (4-10) | 7 (4-10) | 8 (5-10) | 0.43 |
| PVL [n (%)] | 187 (41.74) | 166 (42.13) | 21 (38.89) | 0.66 |
| PM-Rhythm [n (%)] | 83 (18.78) | 72 (18.46) | 11 (21.15) | 0.71 |
| Stroke [n (%)] | 10 (2.2) | 10 (2.51) | 0 (0) | 0.63 |
| MI [n (%)] | 1 (0.22) | 1 (0.25) | 0 (0) | >0.99 |
| **Follow up** |  |  |  |  |
| Follow up time [median (IQR), months] | 38 (28.25-48) | 38 (27.5-48) | 41 (36-48) | 0.21 |
| Death [n (%)] | 169 (37.22) | 151 (37.84) | 18 (32.73) | 0.55 |
